# Immobilization of Naringinase from *Aspergillus Niger* on a Magnetic Polysaccharide Carrier

**DOI:** 10.3390/molecules25122731

**Published:** 2020-06-12

**Authors:** Joanna Bodakowska-Boczniewicz, Zbigniew Garncarek

**Affiliations:** Department of Biotechnology and Food Analysis, Wroclaw University of Economics and Business, 53–345 Wroclaw, Poland; joanna.bodakowska@ue.wroc.pl

**Keywords:** naringinase, immobilization, magnetic carrier, dextran aldehyde

## Abstract

Naringinase is an enzymatic complex used in the deglycosylation of compounds with a high application potential in the food and pharmaceutical industries. The aim of the study was to immobilize naringinase from *Aspergillus niger* KMS on a magnetic carrier obtained on the basis of carob gum activated by polyethyleneimine. Response surface methodology was used to optimize naringinase immobilization taking into account the following factors: pH, immobilization time, initial concentration of naringinase and immobilization temperature. The adsorption of the enzyme on a magnetic carrier was a reversible process. The binding force of naringinase was increased by crosslinking the enzyme with the carrier using dextran aldehyde. The crosslinked enzyme had better stability in an acidic environment and at a higher temperature compared to the free form. The immobilization and stabilization of naringinase by dextran aldehyde on the magnetic polysaccharide carrier lowered the activation energy, thus increasing the catalytic capacity of the investigated enzyme and increasing the activation energy of the thermal deactivation process, which confirms higher stability of the immobilized enzyme in comparison with free naringinase. The preparation of crosslinked naringinase retained over 80% of its initial activity after 10 runs of naringin hydrolysis from fresh and model grapefruit juice.

## 1. Introduction

Naringinase is an enzyme complex showing dual activity: α-l-rhamnosidase (EC 3.2.1.40) and β-d-glucosidase (EC 3.2.1.21). Due to the ability to deglycosylate compounds containing α-rhamnose or β-glucose at the end of the molecule, many natural glycosides can be substrates for naringinase [1]. These include, among others: naringin, rutin, quercetin, hespedrin, neohesperidine, diosmin, myricitrin, monoterpenes and some saponins including ginsenosides [2,3,4,5,6,7]. Naringinase is used, among other applications, in bioconversion of rhamnolipids, for production of glycopeptide antibiotic and sweeteners. The products of naringin hydrolysis, including isocercetin, rhamnose, prunin and naringenin, are the base material for the synthesis of many substances. The specific reaction of naringinase is the hydrolysis of naringin. Naringin is a dominant bioflavonoid found in citrus fruit, mainly in grapefruit, giving it a bitter, characteristic taste [8]. Naringinase plays an important role mainly in citrus fruit processing, reducing the intensity of its bitter taste.

However, considering that naringinase is not adapted to functioning in extreme industrial conditions and the use of its free form hinders enzyme recovery from the reaction medium, immobilized naringinase is necessary to obtain stable and easily recycled preparations.

Due to the variety of biocatalysts and the wide possibilities of their use after immobilization, there is no single, optimal method of enzyme immobilization. Recently, a special interest of researchers has been focused on the immobilization of enzymes on carriers with magnetic properties [9,10,11,12,13,14,15]. The biggest advantage of their use is the quick and easy separation of the enzyme from the reaction medium by means of a magnetic field. This is particularly important when the process takes place in the presence of insoluble substances, such as in grapefruit juice [14,15,16]. Among the methods of immobilizing enzymes on magnetic carriers, covalent binding and physical adsorption are common [14]. The most common material for obtaining magnetic carriers is iron oxide (Fe_3_O_4_).

Carriers of natural origin are often used to immobilize enzymes. For this purpose, proteins, porous glass, silica gel and polysaccharides are used [17,18]. Polysaccharide carriers, thanks to the presence of many chemically active groups, relatively large specific surface area and significant sorption capacity, are a good matrix for enzyme immobilization [19]. Among natural carriers for immobilization of naringinase, the following were used: chitin [20], chitosan [21], alginate [22,23,24,25,26,27,28].

Carriers used for enzyme immobilization are often modified to increase the efficiency of enzyme binding to the matrix [17,29]. The carrier containing aldehyde functional groups is exposed to ionic polymers with NH_2_ groups, such as polyethyleneimine (PEI). Alternatively, a polymer skeleton containing amine groups is activated by means of a reagent containing aldehyde groups, e.g., glutaraldehyde (AG) [30]. Regardless of the immobilization method, glutaraldehyde is the most commonly used compound modifying the surface of the carrier [16,31,32,33,34,35].

In the case of immobilization of enzymes by adsorption, additional crosslinking of the enzymatic protein with a carrier is often used to increase the stability of the obtained system. The most commonly used compound for this purpose is glutaraldehyde [33]. Another compound additionally binding enzymes with a carrier is dextran aldehyde [30,36].

There are only a few articles on the immobilization of naringinase on magnetic carriers in the literature [16,37,38,39], including one on a magnetic polysaccharide carrier [21]. However, most of these studies lack information on the thermodynamic characteristics of the immobilized enzyme. Only Trabizadeh et al. [39] have developed full thermodynamic characteristics of naringinase from *A. aculeatus*, immobilized in the form of naringinase aggregates with amine groups of lysine bound to Fe_3_O_4_. However, the longest half-life of such an immobilized enzyme was just over 8 h. Therefore, it seems advisable to develop other, milder methods of immobilization of this enzyme in relation to naringinase in order to increase its thermal stability.

The aim of the study was to immobilize naringinase from *A. niger* KMS, on a magnetic, polysaccharide carrier and crosslinking bound naringinase with dextran aldehyde. The crosslinked enzyme was used for hydrolysis of naringin contained in fresh and model grapefruit juice. For the immobilized enzyme, the stability depending on the process temperature as well as the activation energy and the half-life depending on the temperature were determined.

## 2. Results and Discussion

### 2.1. Immobilization of Naringinase by Adsorption on Magnetic Carrier

To immobilize the naringinase, a magnetic carrier obtained on the basis of polyethyleneimine activated locust bean gum was used [40]. The carriers obtained on an Fe_3_O_4_ basis are biocompatible, nontoxic and do not show magnetic properties after the removal of the external magnetic field [14]. Magnetite particles may oxidize or be chemically unstable in an acidic environment. Therefore, the coating of a protective layer, e.g., polysaccharides, on the magnetic particles is necessary to maintain their stability [29]. Locust bean carriers, due to the presence of many chemically active groups, relatively large specific surface area and significant sorption capacity, are a good matrix for enzyme immobilization [19]. The Fe_3_O_4_ locust bean complex was modified by PEI to increase the efficiency of enzyme binding to the matrix.

The average particle size of the carrier used, determined by the laser diffraction method, was 117.29 ± 12.31 μm. In the preliminary studies, naringinase was immobilized from the liquid crude preparation with activity 1.253 ± 0.041 µmol·min^−1^·mL^−1^.

As a result of immobilization, 2.113 mg of enzymatic protein per 1 g of the carrier was bound. This made it possible to obtain the immobilized naringinase preparation with activity equal to 3164 μmol·min^−1^·g^−1^ of the carrier. The efficiency of naringinase immobilization on the carrier obtained on the basis of polyethyleneimine activated locust bean gum was 7.6% in relation to the total activity used and 4.5% calculated as the amount of protein.

Using micro- and nanoparticles as immobilization supports is achieving increasing importance. Magnetic nanoparticles have been used in enzyme immobilization owing to their unique properties, such as minimizing diffusion problems. Another essential factor to take advantage of magnetic nanoparticles is easy separation under external magnetic fields. Nanoparticles have a high surface area and a large surface-to-volume ratio [41,42,43].

According to Virgen-Ortíz et al. [44], the adsorption of enzymes on the polyethyleneimine (PEI) activated carrier is quite strong because PEI has many cationic groups at various distances, which can be adjusted to the distances between the active enzyme groups. In addition, the polymer has a random coil structure and does not force distortion of the enzyme through the formation of multiple bonds. The carrier activated by polyethyleneimine, as opposed to two-dimensional adsorption of standard matrices, allows for three-dimensional absorption of the enzyme [45]. In order to check the strength and the way of binding the enzyme to the carrier, a desorption test was carried out.

### 2.2. Desorption

The carrier was regenerated by desorption of the naringinase preparation from its surface using a 10% (*w*/*v*) aqueous solution of sodium chloride or 4% (*v*/*v*) aqueous surfactant preparation, whose main components were anionic surfactants.

Table 1 shows the activity of naringinase immobilized by adsorption as a result of its desorption from the carrier surface. After desorption with a 10% (*w*/*v*) aqueous solution of NaCl, the carrier showed only 7.6% of the initial activity of the immobilized naringinase preparation. Desorption with a 4% (*v*/*v*) aqueous surfactant solution was less effective; naringinase remaining on the surface of the carrier showed over 40% of its initial activity. The analysis of the results of research on protein desorption from the surface of the carrier shows that the use of a 10% (*w*/*v*) sodium chloride solution for this purpose is an effective method of regeneration of a polysaccharide magnetic carrier and indicates the ionic nature of the enzyme binding to the carrier.

The carrier which was subjected to the desorption with sodium chloride solution was used to immobilize a new batch of the enzyme. On the carrier obtained as a result of desorption with sodium chloride solution, a new batch of the enzyme was successfully immobilized again. The activity of naringinase immobilized in this way was 3.300 ± 0.152 µmol·min^−1^·g^−1^ of the carrier, which was 4.4% higher than after the initial immobilization. The desorption of naringinase immobilized on the PEI-activated magnetic support indicates that naringinase was adsorbed via ion exchange. Similarly, pectinase was immobilized on a PEI-modified polymer support [30].

The immobilization of the enzyme on the carrier activated by polyethyleneimine is a reversible system, so the carrier can be reused. Due to the use of adsorption as a technique of enzyme immobilization, the carriers after protein desorption can be reused, thus reducing production costs [46].

Other authors have also examined the possibility of recovering the carrier in order to immobilize a fresh portion of the enzyme. Then, 92% of β-glucosidase immobilized on an agarose carrier and stabilized with polyethyleneimine was recovered from the matrix with a 200 mM salt solution [47].

### 2.3. Optimization of the Process of Naringinase Immobilization on a Polysaccharide Magnetic Carrier

In the next stage of research on the immobilization of naringinase, the influence of particular parameters of the immobilization process, i.e., pH, immobilization time, initial concentration of naringinase preparation and immobilization temperature, on the activity of obtained biocatalytic systems were determined. For this purpose, the experiment was planned according to Box-Wilson’s central composition plan (Table 2).

In the conducted experiment, a solid preparation of the enzyme with an activity of 816 µmol·min^−1^·g^−1^, obtained from the culture of *A. niger* KMS, was used.

The results of the experiment conducted in accordance with the Box-Wilson composition plan were used to determine the coefficients of the square model regression of the response surface. Only statistically significant (*p* < 0.05) coefficients were taken into account in the equation:

(1) $$f=15.50-0.556x_{1}-2.1\left(1\right)62x_{1}^{2}+1.757x_{2}-2.857x_{2}^{2}-1.756x_{3}^{2}-2.479x_{4}-2.608x_{4}^{2}-4.067x_{1}x_{2}+1.319x_{2}x_{4}$$

where:

- *f*—activity of immobilized naringinase (µmol min^−1^ g^−1^ of the carrier);
- *x_1_*—pH (nondimensional values);
- *x_2_*—immobilization time (nondimensional values);
- *x_3_*—concentration of naringinase preparation (nondimensional values);
- *x_4_*—temperature (nondimensional values).

The determination factor R^2^ of this model was 0.98911, which means that over 98% of the variability of immobilized naringinase activity was explained by the model used. The determined equation describing the response surface was used to determine the predictable activity of immobilized naringinase in the analyzed area of variability of the immobilization process parameters. It was also the basis for determining the optimal immobilization parameters, allowing the maximum activity of immobilized naringinase to be obtained. As a result of optimization calculations performed using the MATLAB program, it was found that under the analyzed conditions of the immobilization process, the highest activity of immobilized naringinase equal to 17.00 μmol·min^−1^·g^−1^ of the carrier was obtained after 5.9 h of immobilization of the enzyme from the solution of the enzyme preparation with a concentration of 0.4 g·100 mL^−1^ in 0.01 M buffer with pH 5.06, at 29 °C.

Optimal immobilization parameters allowed us to obtain a naringinase preparation with the activity of 17.06 ± 0.20 μmol·min^−1^·g^−1^ of the carrier. The immobilization efficiency under these conditions was 13.9% calculated based on the total activity of this enzyme used for immobilization and 11.2% calculated based on the protein used.

**Figure 1:**
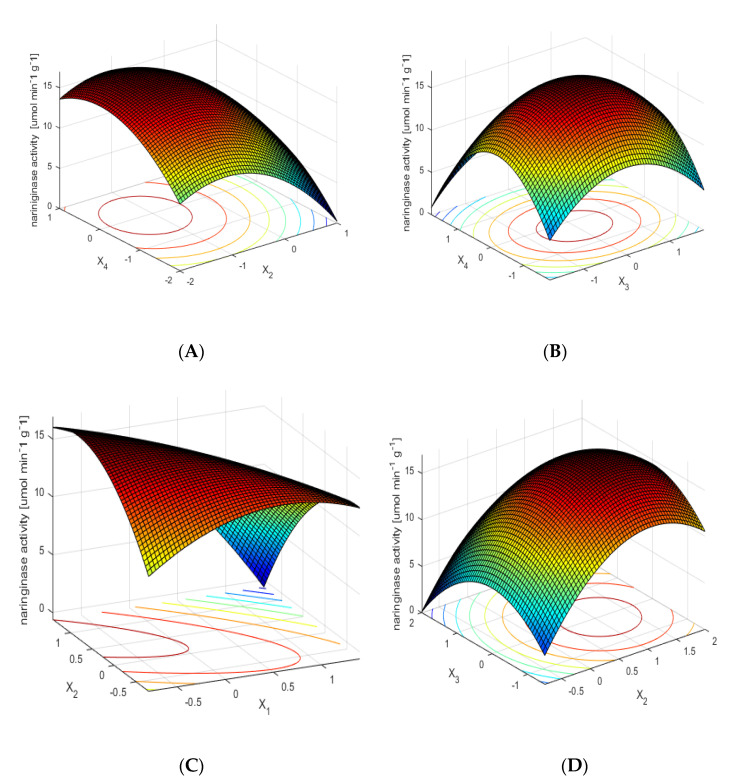
Effect of pH (x_1_), immobilization time (x_2_), protein concentration used for immobilization (x_3_) and immobilization temperature (x_4_) on the activity of immobilized naringinase from *A. niger* KMS. The parameters of the immobilization process were expressed in nondimensional values. Each figure shows the dependence of activity on two factors, with the other optimal values. (**A**) Effect of immobilization time (x_2_) and immobilization temperature (x_4_) on the activity of immobilized naringinase from *A. niger KMS.* (**B**) Effect of protein concentration used for immobilization (x_3_) and immobilization temperature (x_4_) on the activity of immobilized naringinase from *A. niger* KMS. (**C**) Effect of pH (x_1_) and immobilization time (x_2_) on the activity of immobilized naringinase from *A. niger* KMS. (**D**) Effect of immobilization time (x_2_) and protein concentration used for immobilization (x_3_) on the activity of immobilized naringinase from *A. niger* KMS.

Figure 1A shows the dependence of naringinase activity on the time and temperature of the immobilization process of the examined enzyme, with optimal values of the other two parameters. The dependence shows that the highest naringinase activity is obtained as a result of its immobilization at a moderate temperature over a longer time. The short immobilization time and high temperature of the process influence the decrease of immobilized naringinase activity.

The influence of the concentration of the protein used for immobilization and the immobilization temperature on the activity of immobilized naringinase, with optimal values of other parameters, is shown in Figure 1B. The mean values (within the range of variability studied) of temperature and concentration of enzymatic protein used for immobilization allow one to obtain the preparation of immobilized naringinase with the highest activity.

Figure 1C shows the effect of pH and immobilization time on the activity of the immobilized enzyme, with constant optimal values of other parameters. The relation presented in the figure shows that obtaining the high activity of the immobilized enzyme, using low pH values of the solution, requires a longer time of naringinase immobilization.

The influence of immobilization time and protein concentration used for immobilization on the activity of immobilized naringinase, with optimal values of other parameters, is shown in Figure 1D. The highest naringinase activity was obtained with a long immobilization time for the whole analyzed range of protein concentration.

In the studies of other authors, little attention is paid to determining the appropriate conditions of naringinase immobilization and their influence on the activity of the immobilized enzyme. Luo et al. [48], who optimized the parameters of immobilization of crude naringinase from the fermentation broth of *A. niger* FFCC uv-11 on mesoporous silanized silica material, showed that 35 °C was the optimal temperature of the immobilization process of the examined enzyme.

The pH value of the solution used in the immobilization process affects the ionization of functional groups of enzymatic protein [48]. Moreover, the immobilization of naringinase may also be affected by ionization of the carrier caused by the change of polyethyleneimine load [49]. During encapsulation of naringinase in gels, the authors determined the optimal initial pH of the enzyme solution, which, depending on the immobilization method, ranged from 6.0 to 7.0 [50,51]. As a result of immobilization of naringinase from *A. niger* FFCC uv-11 on mesoporous silanized silica material, at pH 5.0, the immobilized enzyme showed 88% of its maximum activity, which it reached at pH 3.5 [48]. The opposite results were obtained in the study on the immobilization of *A. niger* CECT 2088 crude naringinase in polyvinyl alcohol gel [50]. The highest activity of naringinase was obtained as a result of its immobilization from a solution of neutral pH, while significant decreases in activity were observed at acidic and alkaline pH values.

In order to effectively bind naringinase to the carrier, the appropriate immobilization time is often required. Naringinase from *A. niger* KMS strain immobilized on the magnetic polysaccharide carrier showed the highest activity after almost 6 h of its immobilization. Luo et al. [48] observed that when the immobilization time was within 2–4 h, the activity of naringinase increased. However, with immobilization times longer than 4 h, the naringinase activity decreased rapidly. This can be explained by the fact that the longer immobilization time increases the steric hindrance effect between the naringinase molecules or near its active center.

The amount of bound enzymatic protein and the activity of the obtained preparation of the immobilized enzyme often depends on the concentration of the enzyme in the solution used for immobilization. This dependence was noted in the papers by Ribeiro and Ribeiro [28] and Inês Amaro [34]. They demonstrated that with the increase in naringinase concentration, the activity of the immobilized enzyme increases. Vila-Real et al. [51] found that the high efficiency of naringinase immobilization by bioencapsulation observed with lower enzyme concentrations. In the case of the naringinase preparation from *A. niger* KMS immobilized on PEI-activated magnetic polysaccharide carrier, the highest activity was obtained when the concentration of the enzyme in the solution used was 0.4 g per 100 mL.

The immobilization efficiency of naringinase from *A. niger* KMS on the activated polysaccharide carrier under optimal conditions was 13.9% calculated based on the total activity of this enzyme used for immobilization and 11.2% calculated based on the protein used. Similar immobilization efficiency was obtained for pectinase immobilized by a similar method. The adsorption of pectinase on the epoxy carrier activated by polyethyleneimine was carried out with an efficiency of 12% calculated based on the activity of the enzyme [30]. The efficiency of immobilization by the adsorption method at the level of a few to several percent is a typical value of this method of immobilization [52,53,54,55,56,57]. Significantly higher enzyme immobilization efficiencies, including naringinase, are obtained when using other methods, e.g., gel entrapment [28,47,48,54]. The efficiency of naringinase immobilization from *A. niger* CECT 2088 in the polyvinyl alcohol gel was 91.6% calculated based on the total enzyme activity [50]. Similarly, as a result of the immobilization of naringinase in the sol-gel carrier, the immobilization efficiency was 92% in relation to the protein used [51]. However, these immobilization methods do not allow one to remove the used, inactive enzyme from the carrier and reuse it from immobilization.

### 2.4. Crosslinking of Bound Naringinase with Dextran Aldehyde

In the next stage an attempt was made to increase the binding force of the immobilized naringinase, by its additional binding to the carrier with dextran aldehyde. Table 3 shows the results of crosslinking of bound naringinase with dextran aldehyde.

The analysis of data in Table 3 showed that crosslinking of bound naringinase with dextran aldehyde did not significantly affect the activity of the immobilized enzyme.

**Figure 2:**
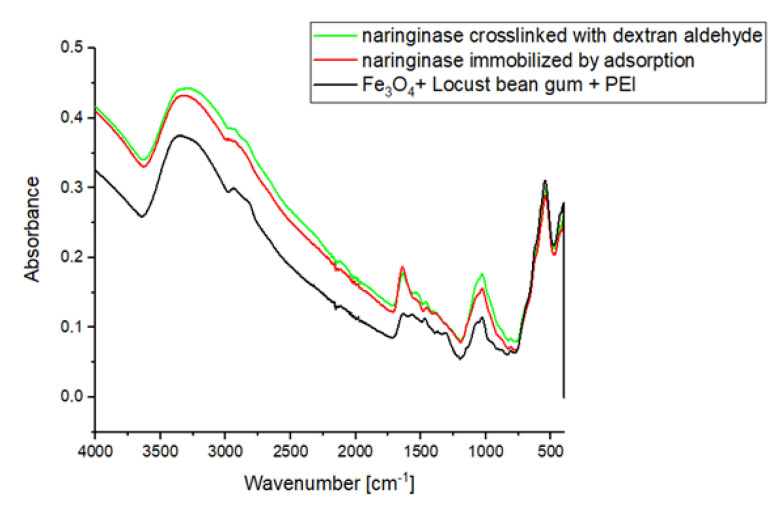
IR ATR spectrum of the magnetic polysaccharide carrier activated by polyethyleneimine, naringinase immobilized by adsorption on the carrier and crosslinking with dextran aldehyde.

In the IR ATR spectrum of the magnetic carrier (Figure 2), a signal is observed, the maximum of which occurs at about 600 cm^−1^ corresponding to Fe-O-Fe stretching vibrations from the carrier’s magnetic core. In the IR ATR spectrum of the carrier, a clear signal is visible in the wavelength range 3360–3297 cm^−1^, which is generated by stretching vibrations of amine groups. In addition, the molecular structure of PEI is characterized by the vibrations of groups C-H (2942 cm–2841 cm^−1^), N-H (1576 cm^−1^), C-H (1465 cm^−1^) and C-N (1356–1000 cm^−1^).

As a result of immobilization of naringinase on the magnetic polysaccharide carrier activated by polysaccharide, an increase in the wide signal absorbance in the range of 3600–3000 cm^−1^ resulting from the presence of stretching vibrations of –OH and –NH_2_ groups of the enzyme is observed. As a result of crosslinking with dextran aldehyde, signals appear on the IR ATR spectrum, which have their maxima at the wavelengths of 1650 and 1530 cm^−1^, corresponding to amide bonds. The presence of amide bonds indicates the formation of Schiff’s alkaline between the amine groups of the enzyme and/or polyethyleneimine and the carbonyl groups of dextran aldehyde.

Additional binding of the enzyme to the carrier with dextran aldehyde has many advantages. First of all, the polymers, due to their molecular size, can cover a large part of the enzymatic protein, and through a small chemical modification of the enzyme, they can easily modify a large area of the protein surface [58]. This makes stabilizing the immobilized enzymes with aldehyde an appropriate strategy for preventing dissociation of enzymatic complexes or their subunits from the carrier surface. This action allows one to improve not only the stability of the enzyme but also the efficiency of the enzyme in the reactions catalyzed by it [58]. Moreover, the reaction between dextran aldehyde and amino groups of enzymes is crucial for the stabilization of immobilized enzymes [36].

The use of PEI-coated carriers ensures strong ionic interaction with the enzyme, avoiding the limitations typical of covalent immobilization [41]. Similarly, the use of dextran aldehyde allows the stabilization of enzyme-carrier interactions by crosslinking, preventing the dissociation of enzymatic subunits [59].

A technique of immobilization of biocatalysts on PEI-coated carriers, additionally stabilized with dextran aldehyde, was used to immobilize several enzymes, such as pectinase [30], lipase [36], uridine phosphorylase [60], thymidine phosphorylase [61], deoxyribosyltransferase [62] and penicillin acylase [63]. Rajdeo et al. [35] effectively immobilized pectinase on a PEI-activated polymer carrier. The enzyme was initially adsorbed on the carrier and then stabilized by binding with dextran aldehyde.

A similar strategy was applied in the case of crude naringinase from *A. niger* immobilized in PEI-activated alginate beads, but in this case, glutaraldehyde was used as a stabilizing agent [64]. Additionally, Ondul et al. [65] immobilized lipase on cotton fabric fibers with adsorbed PEI, additionally stabilized with glutaraldehyde.

Rajdeo et al. [30] used dextran aldehyde, glutaraldehyde and alginate aldehyde to stabilize pectinase. The stabilization of the enzyme-carrier bond with glutaraldehyde resulted in the loss of the enzyme activity, probably due to disturbances in the enzyme active site. Alginate aldehyde did not inactivate the bound enzyme, but also did not contribute to its stabilization. Coating the enzyme with dextran aldehyde resulted in strong binding of pectinase to the polymer, so it could be used several times.

### 2.5. Characteristics of Naringinase from A. Niger KMS

#### 2.5.1. Effect of pH on Naringinase Activity

**Figure 3:**
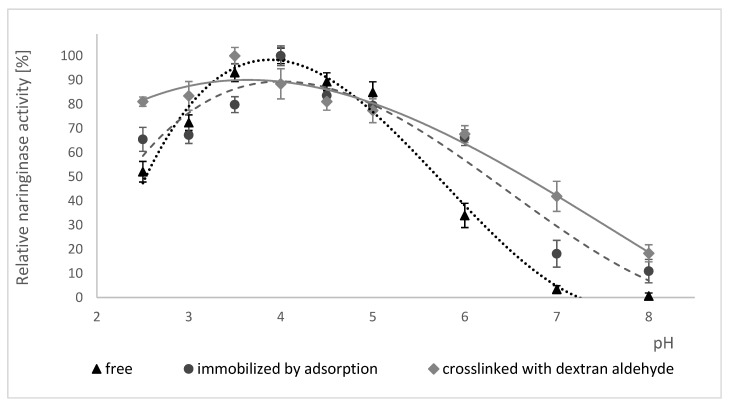
Effect of pH on the activity of free, immobilized and crosslinked naringinase from *A. niger* KMS. Activity of free naringinase (100% = 878 µmol min^−1^ g^−1^ of the preparation); Activity of naringinase immobilized by adsorption (100% = 17.04 µmol min^−1^ g^−1^ of the carrier); Activity of naringinse crosslinked with dextran aldehyde (100% = 19.29 µmol min^−1^ g^−1^ of the carrier). The activity of enzymes was determined at 50 °C, at the pH range 2.5–8.

The influence of pH on the activity of free naringinase, naringinase immobilized by adsorption and naringinase crosslinked with dextran aldehyde is presented in Figure 3. Changes in enzyme activity in the range of pH 2.5–8.0 at 50 °C were studied.

According to the data shown in Figure 3, the immobilization of naringinase on the magnetic carrier activated by PEI did not result in a shift in the optimum pH value of the enzyme, equal to 4.0. The crosslinking of naringinase with dextran aldehyde, on the other hand, resulted in a shift in the optimum pH value from 4.0 to 3.5.

The crosslinked naringinase is characterized by retaining a high activity in a wider pH range than the free naringinase and that immobilized by adsorption. The crosslinked enzyme shows at least 80% activity in the pH range of 2.5 to 3.5.

All studied forms of naringinase showed a high activity at pH 3.0–5.0 and their activity was negligible at alkaline pH, which indicates a higher sensitivity of naringinase to alkaline pH.

The highest activity of crosslinked naringinase at pH 3.5 indicates the possibility of using such an immobilized enzyme in acidic medium as citrus juice. Moreover, the low pH of the solution reduces the possibility of contamination by undesirable microorganisms [66] of substrates, products and an enzymatic reactor [67].

Mishra and Kar [24], Puri and Kalra [68] and Ni et al. [69] reported that the optimal pH value of the free enzyme from *A. niger* is 4.0. Igbonekwu et al. stated that the free naringinase from *A. niger* shows the maximum activity at pH 3.5 [70]. On the other hand, Busto et al. [50] and Luo et al. [48], found that naringinase from *A. niger* is most active at pH 4.5.

Busto et al. [50] showed that the immobilization of naringinase from *A. niger* CECT 2088 in polyvinyl alcohol gel does not change the optimal pH value of 4.5. Similarly, Luo et al. [48] showed that naringinase from *A. niger* FFCC uv-11 immobilized on silica material retains a constant optimal pH value of 4.5.

Awad et al. [64] also noted a decrease in the optimal pH value of naringinase from *A. niger* after its covalent binding on the alginate carrier hardened with polyethyleneimine and glutaraldehyde.

#### 2.5.2. Effect of Incubation in Buffers with Different pH on the Activity of Free, Adsorption-Immobilized and Crosslinked Naringinase

**Figure 4:**
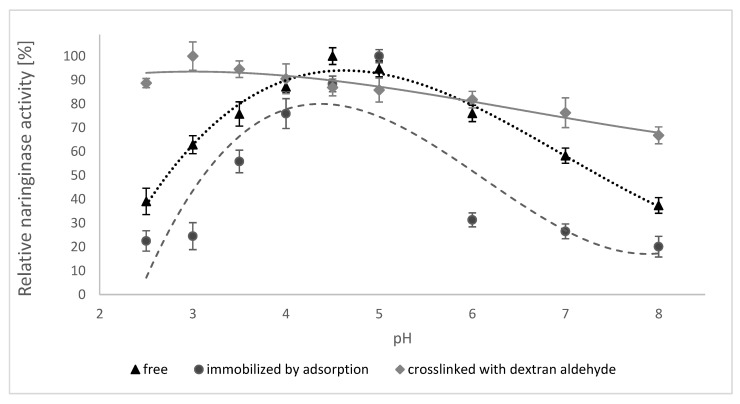
Stability of free, adsorption-immobilized and crosslinked naringinase from *A. niger* KMS depending on the pH of the environment. Activity of free naringinase (100% = 411 µmol min^−1^ g^−1^ of the preparation); Activity of naringinase immobilized by adsorption (100% = 16.72 µmol min^−1^ g^−1^ of the carrier); Activity of crosslinked naringinase with dextran aldehyde (100% = 16.69 µmol min^−1^ g^−1^ of the carrier). The activity of enzymes was determined at 50 °C, pH 4.0.

The results of the pH stability of free, adsorption-immobilized and dextran aldehyde bound naringinase are shown in Figure 4.

The analysis of the data presented in Figure 4 shows that the smallest losses of activity of the free and adsorption-immobilized enzyme were observed at pH 4.5–5.0, while the enzyme crosslinked with dextran aldehyde was stable at pH 2.5–6.0 and maintained over 80% of its activity in this range.

The high stability of naringinase crosslinked with dextran aldehyde at different pH levels in the environment is beneficial for its use in biotechnological processes in the food industry, such as removing the bitter taste of citrus juices.

The literature only provides information on the stability of the free enzyme depending on the pH of the environment. Shanmugaprakash et al. [71] characterized free crude naringinase from *A. brasiliensis* MTCC 1344, found that this enzyme showed high activity after incubation at pH 5.0–7.0, whereas at lower pH values its activity decreased. Purified naringinase preparation from *A. sojae* was shown to be stable at a relatively high pH, from pH 5.0 to 8.0 [72]. There are no reports on the effect of incubation in solutions with different pH on the activity of immobilized naringinase.

#### 2.5.3. Effect of Temperature on Naringinase Activity

**Figure 5:**
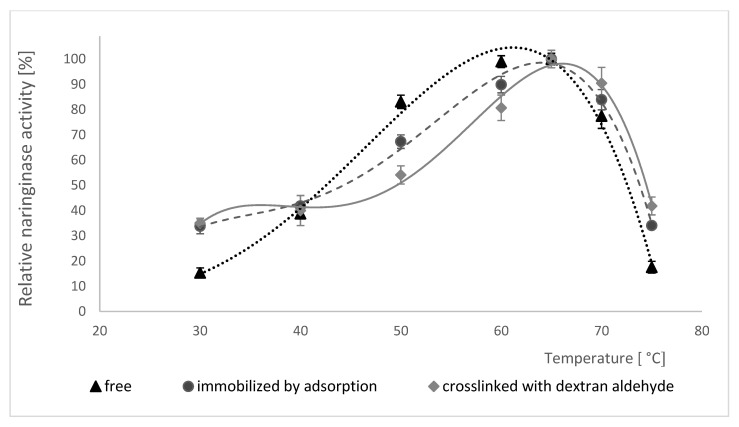
Influence of temperature on activity of free, adsorption-immobilized and crosslinked naringinase from *A. niger* KMS. Activity of free naringinase (100% = 952 µmol min^−1^ g^−1^ of the preparation); Activity of immobilized naringinase by adsorption (100% = 24.81 µmol min^−1^ g^−1^ of the carrier); Activity of crosslinked naringinase with dextran aldehyde (100% = 28.25 µmol min^−1^ g^−1^ of the carrier). The optimum temperature of enzymes was determined at various temperatures (from 30 to 80 °C) at pH 4.0.

Changes in enzymatic activity of free, adsorption-immobilized and crosslinked naringinase were examined in the temperature range from 30 to 80 °C, and the results obtained are shown in Figure 5.

Initially, as the temperature increases, the activity of each of the examined forms of naringinase increases. Free, adsorbed and dextran aldehyde crosslinked enzyme all show the highest activity at 65 °C. Further increase of reaction temperature causes a decrease of enzymatic activity, which is connected with its thermal denaturation. Moreover, the analysis of the data presented in Figure 5 showed that the immobilization and subsequent treatment of naringinase with dextran aldehyde do not affect the change of optimal temperature for the activity of this enzyme compared to its free form.

The immobilization of naringinase, according to some authors, often contributes to the increase in the optimal temperature of this enzyme’s activity. Awad et al. [64] have shown that the covalent binding of naringinase from *A. niger* significantly shifts the optimal temperature from 50 to 70 °C. The immobilization of naringinase from *A. niger* CECT 2088 in polyvinyl alcohol gel also contributed to the increase in the optimal temperature from 60 to 70 °C compared to its native form [50]. Higher optimal temperature of naringin hydrolysis by naringinase increases the possibility of using the crosslinked enzyme on an industrial scale [51]. The use of enzymes in biotechnological processes often encounters the problem of their deactivation under the influence of temperature. As a result of immobilization, a decrease in the sensitivity of biocatalysts to temperature is often observed.

#### 2.5.4. Thermal Stability of Naringinase

**Figure 6:**
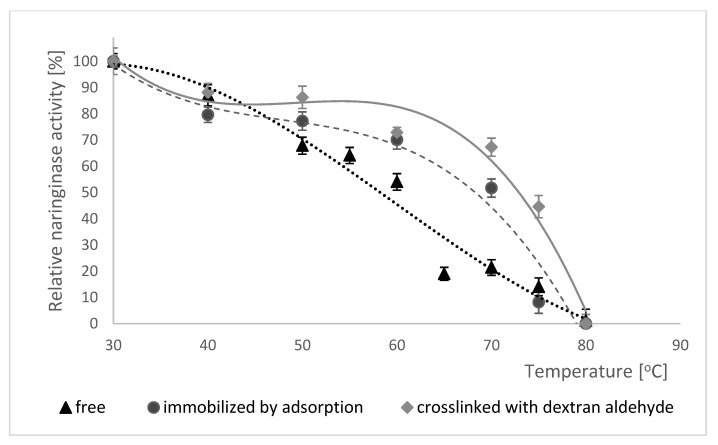
Thermal stability of free naringinase, naringinase immobilized by adsorption and naringinase crosslinked with dextran aldehyde from *A. niger* KMS. Activity of free naringinase (100% = 796 µmol min^−1^ g^−1^ of the preparation); Activity of naringinase immobilized by adsorption (100% = 16.26 µmol min^−1^ g^−1^ of the carrier); Activity of naringinase crosslinked with dextran aldehyde (100% = 16.34 µmol min^−1^ g^−1^ of the carrier). Enzyme activity was determined at 50 °C in buffer with pH 4.0.

The thermal stability of free, adsorption-immobilized and dextran aldehyde coated naringinase was tested by the incubation of all enzyme forms in 0.1 M McIlvaine buffer with pH 4.0 at a temperature from 30 to 80 °C for 60 min. After this time the naringinase activity was determined under standard conditions. The results of the study on the thermal stability of free naringinase, naringinase immobilized by adsorption and naringinase crosslinked with dextran aldehyde are shown in Figure 6.

Analyzing the data presented in Figure 6, a decrease in the activity of free and adsorption-immobilized and crosslinked naringinase was observed along with an increase in temperature.

In the case of enzyme immobilized by adsorption and crosslinked with dextran aldehyde the changes are not as significant as for free enzyme, because naringinase crosslinked with dextran aldehyde shows more than 80% activity after 60 min of incubation in the temperature range of 30–50 °C, and changes in temperature of the reaction environment do not cause rapid decreases in the catalytic activity. This indicates that the immobilized enzyme coated with dextran aldehyde is much more stable than the free and adsorption-immobilized one.

The immobilization of the enzyme on the carrier often limits its conformational changes, stabilizing the three-dimensional structure of the protein [73], due to which the enzyme shows higher resistance to high temperatures [74], which has been confirmed by the authors of several studies.

Awad et al. [64] demonstrated that the immobilization of naringinase from *A. niger* in a biopolymer gel hardened with PEI and AG increased its thermal stability.

### 2.6. Determination of Activation and Deactivation Energy Values and the Naringinase Half-Life

The dependence of the rate of the enzymatic reaction on temperature is described in an Arrhenius equation (Equation (4)). As a result of the linearization of this equation the activation energy (Ea) of free, adsorption-immobilized and crosslinked naringinase was determined.

The determined activation energy values are shown in Table 4.

The immobilization of naringinase on the magnetic polysaccharide carrier caused a decrease in the activation energy to about 28 kJ·mol^−1^. The stabilization of the enzyme with dextran aldehyde did not change significantly the activation energy, compared to the enzyme immobilized by adsorption.

The immobilization of naringinase from *A. niger* KMS by adsorption on the magnetic polysaccharide carrier decreased the activation energy values, thus increasing the catalytic capacity of the tested enzyme. Further coating of the naringinase preparation with dextran aldehyde did not significantly affect the value of its activation energy. The decrease in the activation energy of naringinase from crude *A. niger* CECT 2088 upon immobilized in polyvinyl alcohol gel was also observed by Busto [50].

The activation energy values of the process of thermal deactivation of free naringinase, naringinase immobilized by adsorption and naringinase crosslinked with dextran aldehyde from *A. niger* KMS were also determined (Table 5).

The activation energy of the thermal deactivation process (Ed) is the energy necessary to initiate the denaturation reaction [75]. The activation energy of the thermal deactivation process of the free enzyme (82.996 kJ·mol^−1^) was lower than that of the enzyme immobilized by adsorption (264,751 kJ·mol^−1^) and the enzyme crosslinked with dextran aldehyde (265.069 kJ·mol^−1^). This means that naringinase immobilized and crosslinked with dextran aldehyde was more thermally stable than free enzyme.

Operational stability and half-life times were determined only for crosslinked naringinase because such a preparation showed more excellent stability at elevated temperature and low pH, which is characteristic of grapefruit juice. All these features determine that naringinase immobilized only by adsorption on a magnetic carrier is unlikely to find practical application.

The calculated values of deactivation constants were used to determine the half-life of free and crosslinked naringinase, depending on temperature. The half-life times are presented in Table 6.

As a result of the immobilization and subsequent crosslinking with dextran aldehyde of naringinase preparation from *A. niger* KMS the value of Ed increased almost three times in relation to the free enzyme. These results indicate higher stability of the immobilized enzyme in comparison with free naringinase. This is confirmed by the calculated half-life of the studied forms of naringinase at different temperatures. The enzyme half-life is an important parameter of the economic viability of the bioprocess [28]. In the temperature range studied, i.e., from 25 to 55 °C, the time after which the naringinase activity is reduced to 50% of its initial activity was higher for the immobilized enzyme crosslinked with dextran aldehyde than for the free one. The half-life of naringinase from *A. aculeatus* immobilized in the form of aggregates with amine groups of lysine bound to Fe_3_O_4_ was about 7 h at 50 °C [39].

In the case of naringinase derived from other microorganisms, an increase in the half-life was also observed. The immobilization of naringinase from *Penicillium sp*. on sawdust caused a change in the half-life, at 50 °C, from 90 (free enzyme) to 120 min [33]. Much better stability was demonstrated by naringinase from *P. decumbens*, immobilized by the sol-gel method. After 23 cycles of hydrolysis of naringin solution, at 45 °C, Vila-Real [76] did not find any loss of activity and suggested that, in theory, the half-life of such a system is infinite.

### 2.7. Operational Stability of Naringinase Crosslinked with Dextran Aldehyde

The operational stability, i.e., the maintenance of catalytic activity by the immobilized enzyme preparation when repeating the biotechnological process is one of the most important factors determining the use of the obtained enzymatic complex. The possibility of reuse of naringinase crosslinked with dextran aldehyde was examined by analyzing changes in the enzyme activity, in 10 consecutive reaction cycles.

**Figure 7:**
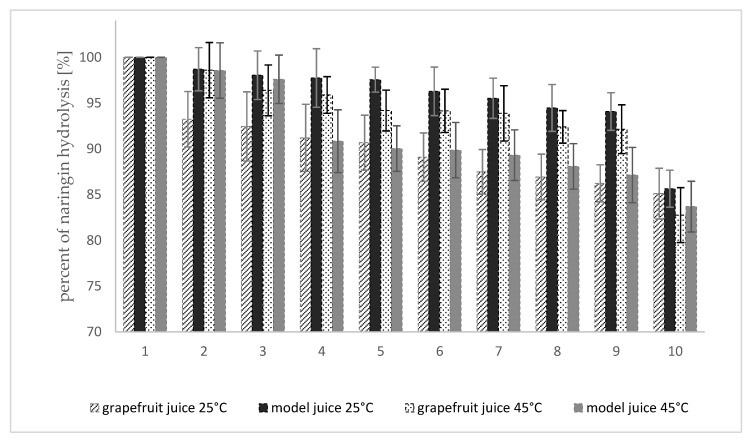
Degree of hydrolysis of naringin in grapefruit juice and model juice by naringinase crosslinked with dextran aldehyde based on 10 consecutive reaction cycles. Freshly squeezed grapefruit juice containing 383 µg·mL^−1^ of naringin.

The results of the study on the change of naringinase activity bound with dextran aldehyde in consecutive reaction cycles are shown in Figure 7.

In the last stage of the study naringinase crosslinked with dextran aldehyde was used for hydrolysis of naringin contained in fresh and model grapefruit juice. Analyzing the determined half-life of crosslinked naringinase as a function of temperature, it was observed that at 45 °C, the calculated half-life is less than 20% shorter than at 35 °C, and at a slightly higher temperature of 50 °C, it is already over 30% shorter. Therefore, it was decided that the hydrolysis of naringin by the enzyme crosslinked with dextran aldehyde would be conducted at 45 °C. Due to the durability of thermolabile components of citrus fruit juices, the hydrolysis reaction was also carried out at 25 °C.

The immobilized naringinase bound additionally with dextran aldehyde, after 10 consecutive reaction cycles carried out at 25 °C, retained over 85% of the initial activity, expressed as the ability to hydrolyze the naringin contained in the model and fresh grapefruit juice (Figure 7). In the case of the reaction carried out at 45 °C, after 10 cycles of use of naringinase bound with dextran aldehyde, the enzyme activity decreased to 82.8% in grapefruit juice and to 83.7% in the model juice, compared to the initial activity.

In the literature, there is little research on the stability of immobilized naringinase in the process of hydrolysis of naringin contained in a juice. The authors of the studies focus only on the hydrolysis reaction of naringin from the model system, often examining the activity of the immobilized enzyme only at optimal temperature. Only Puri et al. [33] demonstrated no visible loss of activity after seven cycles of operation in tangerine juice using covalently bound naringinase on chips covered with glutaraldehyde. Nunes et al. [77] studied the operational stability of naringinase preparation entrapped on electrospun PVA nanofiber of hydrolysis of naringin from the standard solution at two temperatures, i.e., 25 and 45 °C. The researchers showed that naringinase could maintain an average of 68% (at 45 °C) and 100% (at 25 °C) of its initial activity after 188 and 212 h, respectively.

After eight cycles of naringin hydrolysis, at 45 °C, the immobilized enzyme retained slightly over 61% of its initial activity [48]. In another study, magnetic aggregates of naringinase from *A. aculeatus* retained 73% of their initial activity after 10 cycles [39]. Naringinase immobilized in polyvinyl alcohol gel retained over 70% of its initial activity after eight reaction cycles at 30 °C [78].

Naringinase from *A. niger* KMS, immobilized on the magnetic polysaccharide carrier and crosslinked with dextran aldehyde, is characterized by good operational stability compared to other immobilization methods, which creates a great potential for its application to remove the bitter taste and improve the quality of citrus fruit juices as well as other applications.

## 3. Materials and Methods

### 3.1. Materials

Two naringinase preparations obtained from *A. niger* KMS submerged culture were used in the study. The liquid preparation was obtained after separation of mycelium from culture fluid with activity 1.253 ± 0.041 µmol·min^−1^·mL^−1^ and the solid preparation was obtained as a result of concentration by ultrafiltration of postculture fluid and protein precipitation with acetone with activity 816 µmol·min^−1^·g^−1^ [40].

Polysaccharides and activators used for immobilization: locust bean gum from *Ceratonia siliqua*, Sigma; polyethyleneimine 50% water solution, Sigma-Aldrich Chemistry. The main component of carob gum is natural polymer, galactomannan. In addition, locust bean gum consists of other sugars (starch and hemicellulose) and proteins and a small number of lipids [79].

The operational stability studies were conducted using model juice and fresh grapefruit juice. The model juice consisted of 800 µg·mL^−1^ naringin, 0.48% sucrose and 0.025% citric acid [50]. The active acidity of the model juice was 2.93. Fresh grapefruit juice was obtained from grapefruit (Star Ruby variety, country of origin Turkey) bought in a local supermarket. Freshly squeezed grapefruit juice contained 338 µg·mL^−1^ of naringin. The pH of grapefruit juice was 2.92.

### 3.2. Analytical Methods

#### 3.2.1. Determination of Free Naringinase Activity

The activity of free naringinase was determined by the colorimetric method. For this purpose, 5 mg of naringinase preparation was dissolved in 10 mL of 0.9% (*w*/*v*) sodium chloride. A 0.2 mL volume enzyme solution was combined with 0.3 mL of 0.1 M McIlvaine buffer with pH 4.0 and 1 mL of 0.1% (*w*/*v*) naringin solution. The whole was incubated in a thermostat at 50 °C for 30 min. To determine the concentration of naringin in the reaction mixture the Davis method was used [80].

The naringinase activity was expressed in μmol of naringin hydrolyzed for 1 min by 1 g of the enzyme preparation.

#### 3.2.2. Determination of Activity of Immobilized Naringinase and Naringinase Crosslinked with Dextran Aldehyde

To determine the activity of the immobilized or crosslinked naringinase preparation, 1.5 mL of 0.1 M McIlvaine buffer with pH 4 and 3.5 mL of 0.1% (*w*/*v*) aqueous naringin solution were added to a conical flask containing the bound enzyme. The whole was placed on a shaker and mixed with the movement frequency of 150 rpm. The naringin hydrolysis reaction was performed at 50 °C for 30 min. For the evaluation of naringinase activity, Davis’ method was used [80]. The naringinase activity was expressed in μmol of naringin hydrolyzed for 1 min by 1 g of the enzyme immobilized preparation.

#### 3.2.3. Determination of Naringin Content in Juice

The naringin content in the model juice and fresh grapefruit juice was determined by high-performance liquid chromatography using the Perkin-Elmer HPLC system with a UV-VIS detector and autosampler. The separation was carried out on Merck’s Chromolith Performance RP-18e column, using a mobile phase with the composition of acetonitrile: water 23:77, at a flow rate of 1 mL·min^−1^. The detection was carried out at a wavelength of λ = 280 nm.

The application LP-Chrom v. 1.54 was used for the analysis of chromatograms.

#### 3.2.4. Determination of Protein Concentration

The protein content in the postculture liquid, the preparation of obtained naringinase and the enzyme solution used for immobilization was determined by Lowry’s colorimetric method [81]. The absorbance of protein solutions was measured at the wavelength of λ = 750 nm with the Marcel MEDIA spectrophotometer. Then, the absorbance was converted to protein concentration using a standard curve, which was prepared based on beef serum albumin solutions in the concentration range from 0.01 to 1 mg mL.

### 3.3. Immobilization of Naringinase

The immobilization of the naringinase on the magnetic carrier based on the locust bean gum, activated by polyethyleneimine, took place in several stages.

#### 3.3.1. Receiving the Magnetic Carriers

Magnetic microparticles of the carrier covered with polysaccharides were obtained by the modified method given by Wang [46] and described in an earlier paper [82]. In order to obtain magnetite, 3.98 g of ferric chloride (II) tetrahydrate was combined with 6.49 g of ferric chloride (III) and dissolved in 100 mL of water, which allowed Fe^2+^ and Fe^3+^ ions to react in the molar ratio 1:2. The whole was mixed in a round-bottomed flask with a capacity of 500 mL, at room temperature, using a mechanical stirrer at the rotation frequency of 1100 rpm.

Then, 0.5 g of the carob gum dissolved in 20 mL of water was added to the chloride mixture and heated to 65 °C, constantly stirring. After reaching the set temperature with 4M NaOH solution the active acidity of the mixture (pH) was determined at a level of 12 and the mixing was continued for another two hours at 65 °C. At the end of the set time, the mixture was cooled to 20 °C and then its acidity was lowered to pH 7 with 4M acetic acid. The obtained product was drained and washed with distilled water. The obtained carrier microparticles were frozen at −30 °C and excess water was removed by sublimation at a pressure below 10 Pa. The finished carrier was stored at room temperature.

#### 3.3.2. Carrier Activation

The next stage of preparation of the carrier for naringinase immobilization was its activation with polyethyleneimine. The activation of polysaccharide carriers was carried out according to the modified method described by Rajdeo [30] and described in an earlier paper [82]. For this purpose, 1 g of the carrier was weighed into a conical flask with a capacity of 100 mL and then 20 mL of 5% (*v*/*v*) polyethyleneimine solution dissolved in phosphate buffer (0.01 M, pH 7.0) was added. Activation of the carriers was performed on a shaker at the frequency of 150 rpm at 30 °C for 3.5 h.

#### 3.3.3. Measurement of Carrier Particle Size

The particle size measurement of the magnetic polysaccharide carrier activated by polyethyleneimine was performed by laser diffraction of the dry preparation. The tests were performed on the apparatus PSA 1190 LD (Anton Paar, Graz, Austria).

#### 3.3.4. Infrared Spectroscopy

Red-attenuated total reflectance midinfrared (IR-ATRA-MIR, 400–4000 cm^−1^) spectroscopy was used to confirm the binding of polyethyleneimine on the magnetic polysaccharide carrier and the way of binding naringinase to the carrier with dextran aldehyde. The spectra were analyzed using the Origin 2019 program.

#### 3.3.5. Immobilization of Naringinase on the Polysaccharide Magnetic Carrier

The immobilization of naringinase was performed according to the modified procedure described by Jiang [9]. For this purpose, the solution of naringinase preparation from *A. niger* KMS was dissolved in 0.01 M McIlvaine buffer at pH 7, then 150 mg of the carrier was weighed into a conical flask with a capacity of 25 mL and 7.5 mL of the previously prepared solution of naringinase preparation was added. The flasks were shaken at 27 °C for 4 h on a shaker at a movement frequency of 150 rpm.

After the process was completed, the immobilized enzyme was separated from the solution using a neodymium magnet. Then, the obtained biocatalyst was washed several times with 0.01 M phosphate buffer and distilled water with pH 7.

For optimization of the naringinase immobilization parameters, 0.01 M McIlvaine buffer with pH 5.0, 6.0, 7.0, 8.0 and Tris with pH 8.0 and 9.0 were used. The concentration of solid naringinase preparation was 0.13, 0.27, 0.4, 0.53 and 0.67 g per 100 mL. The temperature during this process was 20, 25, 30, 35 and 40 °C and the immobilization time was 2, 3, 4, 5 and 6 h.

#### 3.3.6. Calculation of Efficiency of the Naringinase Immobilization Process

The efficiency of the process of immobilization of naringinase from *A. niger* KMS on the magnetic polysaccharide microparticles was determined in relation to the total activity used and calculated in terms of the amount of protein.

The adsorption efficiency of naringinase from *A. niger* KMS on the surface of the magnetic polysaccharide microparticles, calculated per total enzyme activity, was established by determining the naringinase activity in the solution used for immobilization and the enzyme immobilized preparation and calculated in accordance with the formula:

(2) $$Y_{a}=\frac{Xa}{Xa_{0}}\times 100\%$$

where:

- *Xa*—the difference between the total activity of the enzyme used for immobilization and the activity of the immobilized enzyme;
- *Xa_0_*—the total activity of the enzyme used for immobilization.

A 100% yield was defined as the total activity of naringinase (*Xa_0_*) immobilized.

The efficiency of the process of immobilization of naringinase from *A. niger* KMS on the surface of magnetic polysaccharide microparticles, in relation to the amount of protein, was established by determining the protein concentration in the naringinase solution used for immobilization and in the filtrate after washing the bound naringinase preparation.

The efficiency of the process of naringinase immobilization per protein was calculated from the formula submitted by Vila-Real et al. [57]:

(3) $$Y_{b}=\frac{Xb}{Xb_{0}}\times 100\%$$

where:

- *Xb*—the difference between the total amount of protein used for immobilization and the amount of protein present in the filtrate after washing the immobilized enzyme;
- *Xb_0_*—the amount of protein used for immobilization.

A 100% yield was defined as the total amount of protein (*Xb_0_*) immobilized.

#### 3.3.7. Desorption of the Enzyme from the Surface of the Carrier and its Reimmobilization

To 150 mg of the carrier with immobilized enzyme 10 mL of 10% (*v*/*v*) sodium chloride solution or 4% (*v*/*v*) aqueous solution of surfactant preparation, whose main ingredient were anionic surfactants surfactants (at 15% *v*/*v* sodium lauryl sulfate and sodium laureth sulfate) and amphoteric surfactant (at 5% *v*/*v* caprylyl/capryl glucoside) were added. The mixture was shaken at 30 °C for 60 min on a shaker with a movement frequency of 150 rpm.

The carrier was washed with distilled water and the remaining naringinase activity was determined. Then, on the carrier regenerated in such a way, the tested enzyme was reimmobilized and its activity was determined.

#### 3.3.8. Optimization of the Naringinase Immobilization Process

In order to assess the influence of the immobilization process parameters, i.e., pH, immobilization time, initial enzyme preparation concentration and immobilization temperature, on the activity of immobilized naringinase, an experiment was planned according to Box-Wilson’s central composition plan, with two repetitions at the central point. On the basis of the results of the conducted experiments, a model of square regression of the response area was determined. Only statistically significant coefficients (*p* < 0.05) were taken into account in the formula. The obtained function was used to calculate the maximum activity of immobilized naringinase in the analyzed area of variability of immobilization process parameters. The determination of the optimal point was performed using the hybrid method (combination of genetic algorithm and classical method) using the MATLAB optimization toolbox (MathWorks, MA, USA).

#### 3.3.9. Crosslinking Bound Naringinase with Dextran Aldehyde

In order to bind the enzyme more strongly to the carrier, naringinase was additionally crosslinked with dextran aldehyde. Dextran aldehyde was obtained by the method described by Rajdeo [30].

After the immobilization process was completed, 10 mL of 20% (*v*/*v*) dextran aldehyde was added to the mixture of the remaining solution of the enzyme preparation and the carrier with bound naringinase. The flasks were shaken at 27 °C for 20 h on a shaker with a movement frequency of 150 rpm.

After crosslinking bound naringinase with dextran aldehyde, the obtained complex was separated from a solution using a neodymium magnet and was washed several times with 0.01 M phosphate buffer and distilled water with pH 7. Next, the activity of naringinase by Davis’ method [80] and the amount of protein bound to the carrier by the Lowry method [81] were determined.

### 3.4. Characteristics of a Free, Immobilized and Crosslinked Biocatalyst

#### 3.4.1. Effect of pH on Naringinase Activity

The assessment of the effect of the active acidity of the reaction environment on the activity of free, immobilized and crosslinked naringinase from *A. niger* KMS was made by analyzing the activity of three enzyme forms at different pH values of the mixture. Briefly, 0.1 M McIlvaine buffer (pH 2.5–8.0) was used.

#### 3.4.2. Effect of Incubation in Buffers with Different pH on Naringinase Activity

The stability of free, immobilized and crosslinked naringinase from *A. niger* KMS, depending on pH of the environment, was tested by 15-h enzyme incubation in buffers with different pH, at 4 °C. Then, 0.01 M McIlvaine buffer (pH 2.5–8.0) was applied. The activity of biocatalysts was tested at 50 °C in a buffer with an optimal pH of 4.0.

#### 3.4.3. Effect of Temperature on Naringinase Activity

The effect of temperature on changes in enzymatic activity of free, immobilized and crosslinked naringinase was studied at temperature from 30 to 75 °C. The activity of the tested biocatalysts was determined in 0.1 M McIlvaine buffer with pH 4.0.

#### 3.4.4. Thermal Stability of Naringinase

The thermal stability of free, immobilized and crosslinked naringinase from *A. niger* KMS was tested by incubating all forms of the enzyme at a set temperature for 60 min. The incubation of free, immobilized and crosslinked enzymes was carried out at a temperature from 30 to 80 °C for 60 min. After 60 min the remaining enzyme activity was determined at 50 °C in 0.1 M McIlvaine buffer with pH 4.0.

### 3.5. Determination of Activation and Deactivation Energy and Naringinase Half-Life

The activation energy was determined on the basis of the results of the experiment described in Section 3.4.3. The activation energy of naringinase was determined from the formula:

(4) $$v=k_{0}E\cdot \exp\left(\frac{-E_{a}}{RT}\right)$$

where:

- ν—rate of enzymatic reaction (μmol·min^−1^·g^−1^);
- *k_0_*—constant (min^−1^);
- *E*—enzyme concentration (μmol·g^−1^);
- *E_a_*—activation energy (J·mol^−1^);
- *R*—gas constant (J·mol^−1^·K^−1^);
- *T*—temperature (K).

The value of the activation energy was calculated after the linearization of Equation (4) from the natural logarithm diagram of the initial enzyme reaction rate (ν), at various temperatures (1/T).

In order to calculate the activation energy of the process of thermal deactivation of free, adsorption-immobilized and crosslinked naringinase, an experiment involving continuous hydrolysis of 0.1% (*w*/*v*) naringin solution in 0.1 M McIlvaine buffer with pH 4.0 was carried out. The studies were conducted at temperatures of 40, 45, 50, 55 and 60 °C. The initial activity of the enzyme forms tested was determined and after 2, 5, 10, 24, 48 h of the process. The activation energy of the thermal deactivation process was determined using values of enzyme deactivation constants (*K_d_*), which were calculated using the formula:

(5) $$v=kE_{0}\cdot \exp\left(-K_{d}t\right)$$

where:

- *ν*—rate of enzymatic reaction (μmol·h^−1^·g^−1^);
- *k*—kinetic constant (h^−1^);
- *E_0_*—initial enzyme concentration (μmol·g^−1^);
- *K_d_*—deactivation constant (h^−1^);
- *t*—time (h).

Deactivation constants *K_d_* were calculated for the specified temperature after the linearization of Equation (5) from the natural logarithm diagram of enzyme activity (v) in relation to time (t).

The activation energy of the thermal deactivation process was calculated using the nonlinear estimation method from Equation (6), using the STATISTICA v. 13 program.

(6) $$K_{d}=K_{d0}\cdot \exp\left(\frac{-E_{d}}{RT}\right)$$

where:

- *K_d_*—deactivation constant (h^−1^);
- *K_d0_*—constant (h^−1^);
- *E_d_*—activation energy of the thermal deactivation process (J·mol^−1^);
- *R*—gas constant (J·mol^−1^·K^−1^);
- *T*—temperature (K).

In the next stage of the study the half-life of free, immobilized and crosslinked naringinase was determined, depending on temperature. The half-life of different enzyme forms was calculated from the formula:

(7) $$\tau =\frac{0.693}{K_{d}}$$

where:

- *τ*—half-life (h);
- *K_d_*—deactivation constant (h^−1^).

### 3.6. Operational Stability of Crosslinked Naringinase

The operational stability of the naringinase crosslinked with dextran aldehyde preparation was determined as a result of 10 cycles of hydrolysis of naringin contained in the model and fresh grapefruit juice. Hydrolysis was performed at 25 and 45 °C. In the first cycle, the reaction time was determined, followed by complete hydrolysis of naringin. After the reaction, the carrier with immobilized naringinase was separated using a neodymium magnet and then rinsed with distilled water. Then, the crosslinked naringinase preparation obtained from the previous cycle was added to the fresh portion of juice and the hydrolysis was performed at the same time as in the first cycle. After each hydrolysis cycle, the concentration of naringin remaining in the juice was determined by the HPLC method. Total hydrolysis (100%) of naringin contained in tested juices was taken as the initial activity of crosslinked naringinase.

### 3.7. Statistical Compilation of Results

Measurements of individual values were taken in three repetitions and the results were presented as the arithmetic mean with standard deviation. Microsoft Excel 2013 was used for calculations.

A one-factor analysis of variance was carried out using STATISTICA v. 13. The homogeneity of variance was verified by the Levene and Brown–Forsyth tests and the significance of differences between the means by Duncan’s test. Calculations were performed at a significance level of α = 0.05.

The nonlinear estimation was performed using the least-squares method with the Levenberg-Marquardt algorithm. For this purpose, STATISTICA v. 13 was used.

## 4. Conclusions

The potential use of immobilized enzymes in the food industry requires the use of easily accessible carriers and cheap immobilization methods. The obtained preparation of naringinase immobilized on a magnetic-activated carrier based on locust bean gum and then crosslinked with dextran aldehyde is characterized by high stability in an acidic environment and at high temperatures. The applied immobilization method shifts the optimal pH value for naringinase activity from 4.0 to 3.5 and increases the thermal stability of the immobilized enzyme, which is confirmed by higher activation energy values of the thermal deactivation process and longer half-life periods in comparison with the free form of this enzyme. In addition, the naringinase immobilized and crosslinked with dextran aldehyde from *A. niger* KMS shows good operational stability, supporting its practical application, e.g., in the hydrolysis of naringin contained in grapefruit juice.

## Figures and Tables

**Table 1:** Desorption of enzymatic protein from a polysaccharide magnetic carrier activated by polyethyleneimine.

Desorption Method	Before Desorption	After Desorption	
	Activity (µmol·min^−1^·g^−1^ of a Carrier)	Activity (µmol·min^−1^·g^−1^ of a Carrier)	% of Initial Activity
10% (*w*/*v*) aqueous solution of NaCl	3.160 ± 0.140	0.239 ± 0.015	7.6
4% (*v*/*v*) aqueous surfactant solution	3.160 ± 0.140	1.345 ± 0.052	42.6

**Table 2:** Results of an experiment performed in accordance with Box-Wilson’s central composition plan to determine the effect of immobilization parameters on the activity of immobilized naringinase.

Process Variant	Parameters of the Immobilization Process								Naringinase Activity (µmol·min^−1^·g^−1^ of the Carrier)
	x_1_		x_2_		x_3_		x_4_		
	pH		Immobilization Time		Concentration of Naringinase Preparation		Temperature		
	Coded Levels	(–)	Coded Levels	(h)	Coded Levels	(g·100 mL^−1^)	Coded Levels	(°C)	
1	1	8.0	1	5.0	1	0.53	−1	25.0	5.18
2	1	8.0	1	5.0	−1	0.27	−1	25.0	3.27
3	1	8.0	−1	3.0	1	0.53	1	35.0	4.12
4	−1	6.0	1	5.0	−1	0.27	1	35.0	11.79
5	1	8.0	−1	3.0	−1	0.27	1	35.0	4.25
6	−1	6.0	−1	3.0	1	0.53	−1	25.0	4.57
7	−1	6.0	1	5.0	1	0.53	1	35.0	10.51
8	−1	6.0	−1	3.0	−1	0.27	−1	25.0	4.95
9 (C)	0	7.0	0	4.0	0	0.40	0	30.0	9.50
10	−2	5.0	0	4.0	0	0.40	0	30.0	10.00
11	2	9.0	0	4.0	0	0.40	0	30.0	5.78
12	0	7.0	−2	2.0	0	0.40	0	30.0	0.30
13	0	7.0	2	6.0	0	0.40	0	30.0	7.92
14	0	7.0	0	4.0	−2	0.13	0	30.0	8.41
15	0	7.0	0	4.0	2	0.67	0	30.0	8.62
16	0	7.0	0	4.0	0	0.40	−2	20.0	10.07
17	0	7.0	0	4.0	0	0.40	2	40.0	0.15
18 (C)	0	7.0	0	4.0	0	0.40	0	30.0	8.81

**Table 3:** Effect of dextran aldehyde crosslinking on the activity of bound naringinase.

	Activity (µmol·min^−1^·g^−1^ of Carrier)	Mass of Bound Protein (mg 150·mg^−1^ of Carrier)	Specific Activity (µmol·min^−1^·mg^−1^ of Protein)
Naringinase immobilized by adsorption (IMNA)	Determination before addition of dextran aldehyde		
	17.06 ± 0.20 ^a^	2.528 ± 0.006 ^b^	1.012 ± 0.014 ^c^
Immobilized naringinase after crosslinking	Determination after addition of dextran aldehyde		
	17.04 ± 0.16 ^a^	2.528 ± 0.008 ^b^	1.011 ± 0.006 ^c^

Different letter markings indicate the existence of statistically significant differences at the level of *p* < 0.05.

**Table 4:** Summary of activation energy of free naringinase, naringinase immobilized by adsorption and naringinase crosslinked with dextran aldehyde.

Type of Enzyme	Activation Energy (Ea) (kJ·mol^−1^)
Free naringinase	32.5
Naringinase immobilized by adsorption	28.1
Adsorbed naringinase crosslinked with dextran aldehyde	28.6

**Table 5:** Summary of the activation energy of the thermal deactivation process of free naringinase, naringinase immobilized by adsorption and naringinase crosslinked with dextran aldehyde.

Type of Enzyme	Activation Energy of the Thermal Deactivation Process (Ed) (kJ mol^−1^)
Free naringinase	83.0
Naringinase immobilized by adsorption Adsorbed naringinase crosslinked with dextran aldehyde	264.8 265.1

**Table 6:** Summary of half-life times of free naringinase and naringinase crosslinked with dextran aldehyde depending on temperature.

Temperature (°C)	Half-Life Times (h)	
	Type of Enzyme	
	Free Naringinase	Naringinase Crosslinked With Dextran Aldehyde
35	47.23	68.31
40	24.15	63.00
45	16.00	55.89
50	4.11	45.89
55	1.82	34.65
60	1.62	8.66
62	1.27	4.39
65	1.03	1.94
